# A Novel Enediynyl Peptide Inhibitor of Furin That Blocks Processing of proPDGF-A, B and proVEGF-C

**DOI:** 10.1371/journal.pone.0007700

**Published:** 2009-11-26

**Authors:** Ajoy Basak, Abdel-Majid Khatib, Dayani Mohottalage, Sarmistha Basak, Maria Kolajova, Subhendu Sekhar Bag, Amit Basak

**Affiliations:** 1 Chronic Diseases Program, Regional Protein Chemistry Center, Ottawa Hospital Research Institute, Department of Biochemistry, Microbiology and Immunology, University of Ottowa, Ottawa, Canada; 2 INSERM, UMRS940, Equipe AVENIR. Institut de Génétique Moléculaire, Hospital St-Louis, Paris, France; 3 Université Paris 7, Paris, France; 4 Department of Chemistry, Indian Institute of Technology, Kharagpur, West Bengal, India; 5 Department of Chemistry, Indian Institute of Technology, Guwahati, Assam, India; University of Helsinki, Finland

## Abstract

**Background:**

Furin represents a crucial member of secretory mammalian subtilase, the Proprotein Convertase (PC) or Proprotein Convertase Subtilisin/Kexin (PCSK) superfamily. It has been linked to cancer, tumorgenesis, viral and bacterial pathogenesis. As a result it is considered a major target for intervention of these diseases.

**Methodology/Principal Findings:**

Herein, we report, for the first time, the synthesis and biological evaluation of a newly designed potent furin inhibitor that contains a highly reactive beta-turn inducing and radical generating “enediynyl amino acid” (Eda) moiety. “Eda” was inserted between P1 and P1′ residues of hfurin^98–112^ peptide, derived from the primary cleavage site of furin's own prodomain. The resulting hexadecapeptide derivative inhibited furin *in vitro* with IC_50_ ∼40 nM when measured against the fluorogenic substrate Boc-RVRR-MCA. It also inhibited furin-mediated cleavage of a fluorogenic peptide derived from hSARS-CoV spike protein with IC_50_ ∼193 nM. Additionally it also blocked furin-processing of growth factors proPDGF-A, B and VEGF-C that are linked to tumor genesis and cancer. Circular dichroism study showed that this inhibitor displayed a predominantly beta-turn structure while western blots confirmed its ability to protect furin protein from self degradation.

**Conclusion/Significance:**

These findings imply its potential as a therapeutic agent for intervention of cancer and other furin-associated diseases.

## Introduction

Furin is a crucial member of Ca^+2^-dependent mammalian subtilases collectively known as **Proprotein Convertases (PCs)** or **Proprotein Convertase Subtilisin/Kexins (PCSKs)**. This membrane bound type 1 protease is responsible for tissue-specific endoproteolytic cleavage of a large variety of inactive protein precursors at the general sequence motif (K/R)-(X)n-(R) ↓ (where n  = 0, 2, 4 or 6 and X is usually any amino acid other than Cys), leading to functionally active secretory proteins and polypeptides [1]–[3]. Among the known furin substrates are the precursors of hormones, neuropeptides, growth factors, adhesion molecules, receptors, surface proteins, viral glycoproteins and bacterial toxins [2]. Based on above findings and accumulated studies in the literature, furin has been strongly linked to tumorgenesis, hormonal diseases, neurological dementia and a variety of infectious diseases caused by ebola, avian, Hong Kong, HIV, human SARS corona viruses as well as bacterial pathogenesis due to anthrax toxin, aerolysin etc [4]–[9]. Owing to these results, this enzyme is considered as a potential prognostic factor for several diseases. Therefore many researchers including us became interested in the development of potent and specific furin inhibitors that may possess important biochemical, clinical and therapeutic applications. Until now, several macromolecules and small compound furin inhibitors have been reported in the literature [reviewed in 10,11]. While all macromolecule furin inhibitors are of protein types either endogenous or biologically engineered, the small molecule inhibitors are largely synthetic peptide, peptidomimetic or fully non-peptide compounds [12]–[27]. Owing to increased stability, enhanced bioavailability, improved drug like property and easy accessibility by synthetic means, small molecule inhibitors are always preferred over proteins for therapeutic use [28]. Among the various inhibitor design strategies so far reported, the prodomain approach attracted most attention because of its effectiveness, versatility and sometimes enhanced selectivity [18], [26]. Besides this approach, incorporation of non-cleavable pseudo peptide bond [29] or unnatural amino acid [30] at P1-P1′ site of a potent peptide substrate based on prodomain or physiological protein sequence has also been used successfully to design inhibitors of PC enzymes. Based on somewhat similar idea, herein, we report for the first time, a new and innovative strategy for the design of a potent furin inhibitor. Our approach was primarily based on a specially constructed unnatural amino acid called “Eda or enediynyl amino acid” and its incorporation at the cleavage site of prodomain sequence of furin whose primary role is to regulate the protease activity by providing proper folding after binding. We show that incorporation of this highly reactive beta-turn inducing aromatic “Eda” moiety at the scissile P1-P1′ amide bond of a prodomain peptide of human furin led to a potent furin-inhibitor with inhibition constant K_i_ and IC_50_ in low nM ranges. For many years, enediynes and their derivatives were known to bind and cleave DNA especially of cancer cells through their oxidative actions. This occurred via *in situ* generation of bis-radicals by Bergman cyclo-aromatization reaction [31]. The ease of this cyclization depends on the nature and structure of enediynes [32]–[35]. Thus cyclic diynes of 8–10 member size as well as aza-enediynes have been shown to efficiently undergo Bergman cyclization under the induction of light, metal ions or elevated temperature due to their low activation barrier [reviewed in 32]. Due to this unique ability to cyclize and produce reactive bis-radicals, several enediyne derivatives have been designed as reactive species especially for DNA degradation [36]–[39]. So far several enediynes of both synthetic and fungal metabolite origins have been shown to display strong antitumor activity [31], [40]. Subsequently a few of these compounds have been approved as anti-cancer drugs. In a recent study Jones *et al.* examined the viability of proteins as targets of thermally and photo-activated enediyne derivatives and confirmed at the molecular level. They have shown that specifically designed enediyne derivatives can degrade proteins under suitable conditions [41]–[43]. Based on this observation, we hypothesize that such functional derivatives may also interact with catalytically active proteins such as protease like furin and thereby regulate its enzymatic and functional activity. For efficient binding with the target enzyme furin, we propose that such above moiety must carry a furin recognition peptide sequence. Thus we prepared a novel enediynyl peptide using a combination of organic and peptide chemistry and demonstrated its potent furin inhibitory activity both *in vitro* and cellular models. We first described the synthesis of Fmoc-protected “Eda” and then incorporated it into a furin substrate namely hfurin^98–112^ peptide at its furin cleavage site. The biochemical study showing anti-furin activity of this peptide analog towards a fluorogenic peptide substrate as well as physiological precursor proteins such as growth factors proPDGF-A, B and proVEGF-C are described in the present study.

## Results

### Inhibition of Furin, PC5 and PC7 by Aromatic Enediyne Compounds

**Figure 1:**
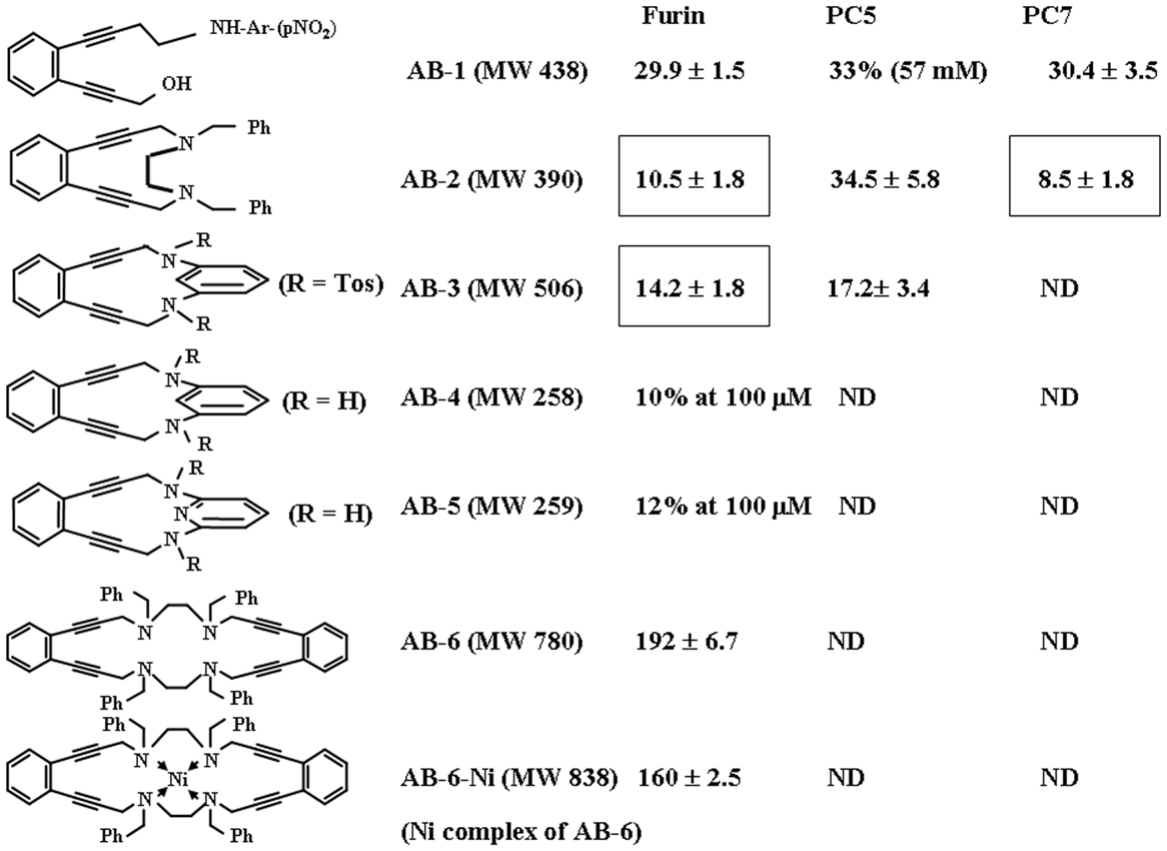
List of synthetic aromatic enediyne derivatives and their effects on protease activities of Proprotein Convertases Furin, PC5 and PC7. The table shows the IC_50_ values for inhibition of recombinant proprotein convertases furin, PC5 and PC7 by various aromatic enediyne compounds. The enzyme assay was carried out by using the fluorogenic peptide substrate Boc-RVRR-MCA (50 µM final concentration) as described in [27]. ND  =  not determined.

Most aromatic enediyne compounds are chemically highly reactive and can easily generate upon heating reactive bis-radical species *via* Bergman cyclo-aromatization reaction [31], [32]. It is a possibility that these reactive intermediates may then react with one or more of the catalytic amino acid residues His, Ser or Asp of PC-enzymes leading to chemical modifications of latter. This notion is supported by several studies involving interactions of amino acids either free or within protein sequences [41]–[46]. Based on this rationale we proposed that such types of molecules may therefore inhibit the protease activities of these enzymes. To test this hypothesis, we first synthesized several aromatic enediyne derivatives [44] and examined *in vitro* their effects on proteolytic activities of three key members of PC-family enzymes namely furin, PC5 and PC7. *In vitro* enzyme assay of PCs against Boc-RVRR-MCA substrate (50 µM), in the absence and presence of various synthetic aromatic enediyne compounds (Fig. 1) indicated weak to modest inhibitory activities of these compounds. Among the PCs examined, the most potent inhibitory activity was noted against PC7 for compound AB-2 which displayed an IC_50_ value of 8.5 µM. For furin, the highest inhibitory activity was also noted with AB-2 with IC_50_ value of 10.5 µM. Except for minor protease activating effect of AB-3 towards PC7, all other enediyne compounds exhibited inhibitory effects towards furin, PC5 and PC7 with IC_50_ values ranging from 8.5 to 160 µM depending on the nature of the enzyme and the enediyne compound used. *This is the first report of PC-inhibitory activity of an enediyne compound*. This finding prompted us to the idea of incorporating an enediyne function within a peptide sequence which may enhance the potency and selectivity of furin inhibition. To accomplish this, we designed and synthesized an enediynyl amino acid (Eda) derivative with its amino terminal protected by Fmoc (9H-fluoren-9-ylmethoxy carbonyl) group so that it can be directly used in solid phase peptide synthesis.

### Synthesis of Fmoc-Eda (I)

**Figure 2:**
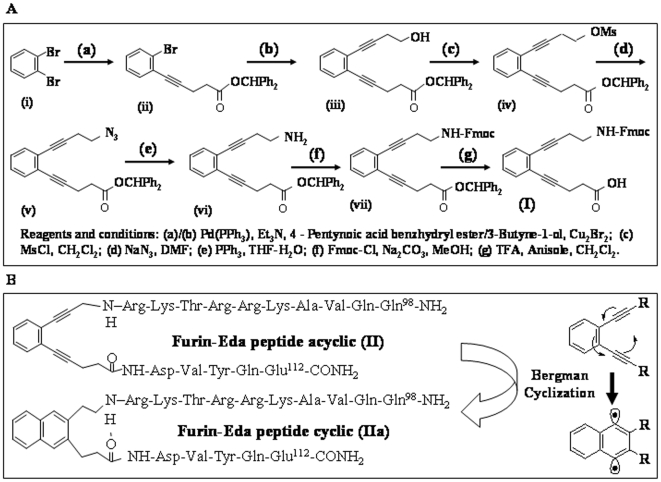
Design of enediyne-based furin inhibitor. Figure 2A. Scheme for chemical synthesis of Fmoc protected enediynyl amino acid (Eda). The reagents used for coupling or chemical reaction in various steps are shown at the footnotes of the scheme. Figure 2B. Design of β-turn mimetic furin inhibitor based on enediynyl amino acid (Eda) and profurin^98–112^. The figure (left panel) shows the structures of furin-Eda peptide (II) and its cyclized form (IIa) following Bergman cyclo-aromatization reaction. The right panel shows the mechanism and electron transfer of Bergman cyclization reaction with the formation of a bis radical intermediate. R  =  Any group; the dotted line indicates hydrogen bonding.

Synthesis of Fmoc-Eda (I) was accomplished by adopting he chemistry similar to that earlier reported by us [44], [47]. The multiple steps involved in the synthesis are shown schematically in self explanatory way in Fig. 2A. All synthetic details and procedure were described under Materials and Methods section.

### Peptide Selection for Incorporation of Eda

**Figure 3:**
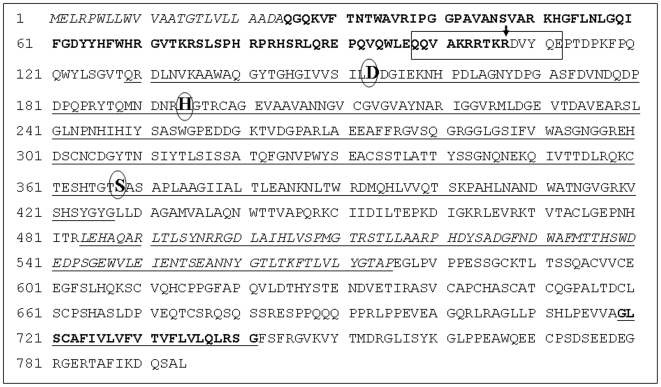
Complete amino acid sequence of human preprofurin. Various domains are highlighted as indicated below: Underlined residues (129–427): Peptidase S8 (Subtilase) domain, (full catalytic domain of hfurin is considered as 108–438); Residues in italics (1–24): Signal peptide; Bold residues (25–107): Prosegment; Residues with underlined italics (484–575): P-domain; Residues with bold underlined (719–741): Transmembrane. The catalytic residues Asp^153^, His^194^, Ser^368^ are shown in bold larger fonts within circles. The peptide segment shown within the box was used for incorporation of “Eda” moiety.

For incorporation of Eda unit, we selected the sequence (98–112) from human furin prodomain (shown within a box in Fig. 3). that comprises its autocatalytic primary cleavage site Arg-Lys-Thr-Arg-Arg^107^↓Asp-Val Various studies have shown that such a peptide bond is efficiently cleaved by furin (shown by vertical arrow) in both *in vitro* and *ex vivo* conditions [11], [18], [26]. In addition, this segment of furin pro-region has been found to be highly conserved across the species [26].

### Synthesis, Purification and Characterization of Furin-Eda-Peptide (II)

**Figure 4:**
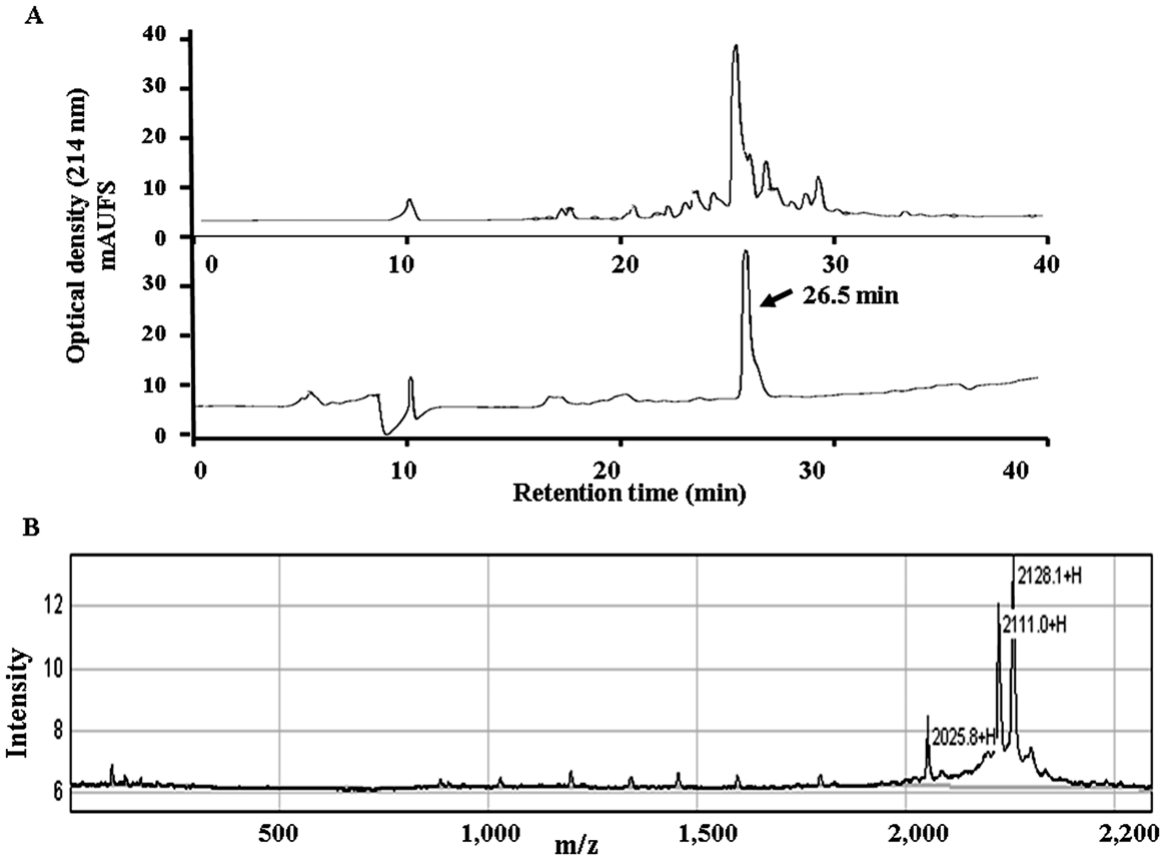
Purification and characterization of furin-Eda peptide. Figure 4A. RP-HPLC chromatograms of furin-Eda-peptide (II). Upper panel: HPLC for crude material obtained directly from solid phase synthesis (using C_18_-semi preparative column), lower panel: HPLC for purified material (using C_18_ analytical column). mAUFS  =  milli absorbance units full scale. Figure 4B. SELDI-tof mass spectrum of purified furin-Eda-peptide (II). It shows major peaks at m/z 2111 and 2118 for (M+H)^+^ and (M+H+oxygen)^+^ ions respectively.

The above furin-Eda-peptide (II) contains a highly reactive β-turn inducing unnatural Eda-amino acid inserted between the scissile **Arg-Asp** amide bond. It is likely that such a peptide analog will be sensitive to changes in pH, metal ion concentrations and other environmental conditions such as UV light [32] that may lead to the formation of a cyclic derivative (IIa) (Fig. 2B) via Bergman cyclo-aromatization reaction. Such chemical transformation and modification may induce protease inhibitory activity to the peptide derivative. Peptide (II) was synthesized by HATU/DIEA (Diisopropyl ethyl amine) mediated solid phase Fmoc chemistry [18], [26] and purified by RP-HPLC (Fig. 4A, upper panel) using a C_18_ semipreparative column. The peak eluting at Rt  = 26.5 min was collected and analyzed by a second HPLC-run on an analytical C_18_ column when it exhibited a single peak at nearly identical retention time showing its high degree of purity (Fig. 4A, lower panel). The identity of the peptide was further confirmed by SELDI-tof mass spectrometry which displayed a peak at m/z  = 2111 in consistent with its calculated molecular weight (MW  = 2110) (Fig. 4B). The second peak observed at m/z  = 2128 may be due to the formation of an oxidized form of II, since it is ∼16 mass unit higher than the molecular ion. However other explanation may also exist.

### Furin Inhibition by Eda Peptide (II)

**Figure 5:**
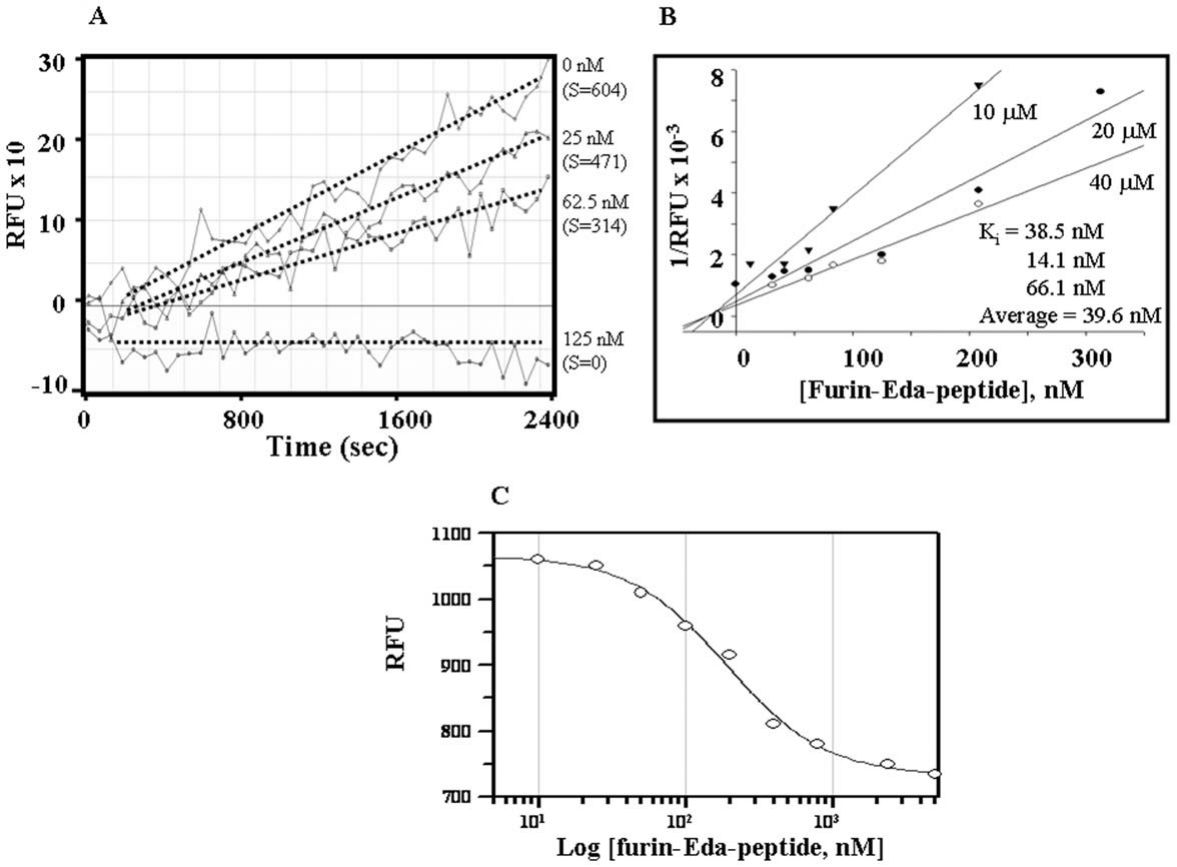
Furin inhibition by furin-Eda peptide. Figure 5A. Progress curves showing inhibition of furin activity by furin-Eda-peptide (II). The assay was conducted using Boc-RVRR-MCA (20 µM) as fluorogenic substrate. Figure 5B. Dixon plots showing inhibition of furin activity by furin-Eda-peptide (II). The inhibition was studied using three different concentrations (as indicated) of Boc-RVRR-MCA fluorogenic substrate (*see* *Materials and Methods* *section for details*). RFU  =  Raw fluorescence unit. Figure 5C. Inhibition of furin-mediated cleavage of hSARS-CoV fluorogenic peptide by furin-Eda-peptide (II). Furin-Eda-peptide blocks furin cleavage of intramolecularly quenched fluorogenic peptide hSARS-CoV spike^754–766^ Abz-AEQDRNTR^761^⇓ EVFAQ-Tyx-A (Abz  = 2-Amino benzoic acid, fluorescent group and Tyx  = 3-Nitro tyrosine, fluorescence quench group).

Both progress curve and stopped-time assays with the fluorogenic substrate Boc (butyloxy)-RVRR-MCA (4-methyl coumarin 7-amide) (Figs. 5A and B) showed that Eda-peptide (II) inhibited furin activity *in vitro* with a high degree of potency. The progress curve assay (Fig. 5A), indicated that 125 nM of (II) was able to block furin activity almost completely against the above substrate (20 µM). As measured by the slope (s) of each curve shown within parenthesis in the graph, this effect was found to be concentration-dependent. The measured IC_50_ value was found to be ∼70 nM. Furthermore, Dixon plots based on stop-time assay at three different substrate concentrations 10, 20 and 40 µM, (Fig. 5B) indicated an inhibition constant (K_i_) of 39.6 nM for furin inhibition by (II). The pattern of the graph also confirmed the competitive nature of inhibition suggesting the binding of Eda-peptide with the catalytic domain of furin. Using another substrate namely the intramolecularly quenched fluorogenic peptide **(Abz-AEQDRNTR^761^↓EVFAQ-Tyx-A)**, where Abz  = 2-amino benzoic acid, Tyx  = 3-nitro tyrosine) derived human SARS corona virus spike protein [8], we noted that (II) blocked its processing with measured IC_50_ value being ∼193±12 nM (Fig. 5C).

### Effect of Furin-Eda-Peptide on proPDGF-A and proVEGF-C Processing

**Figure 6:**
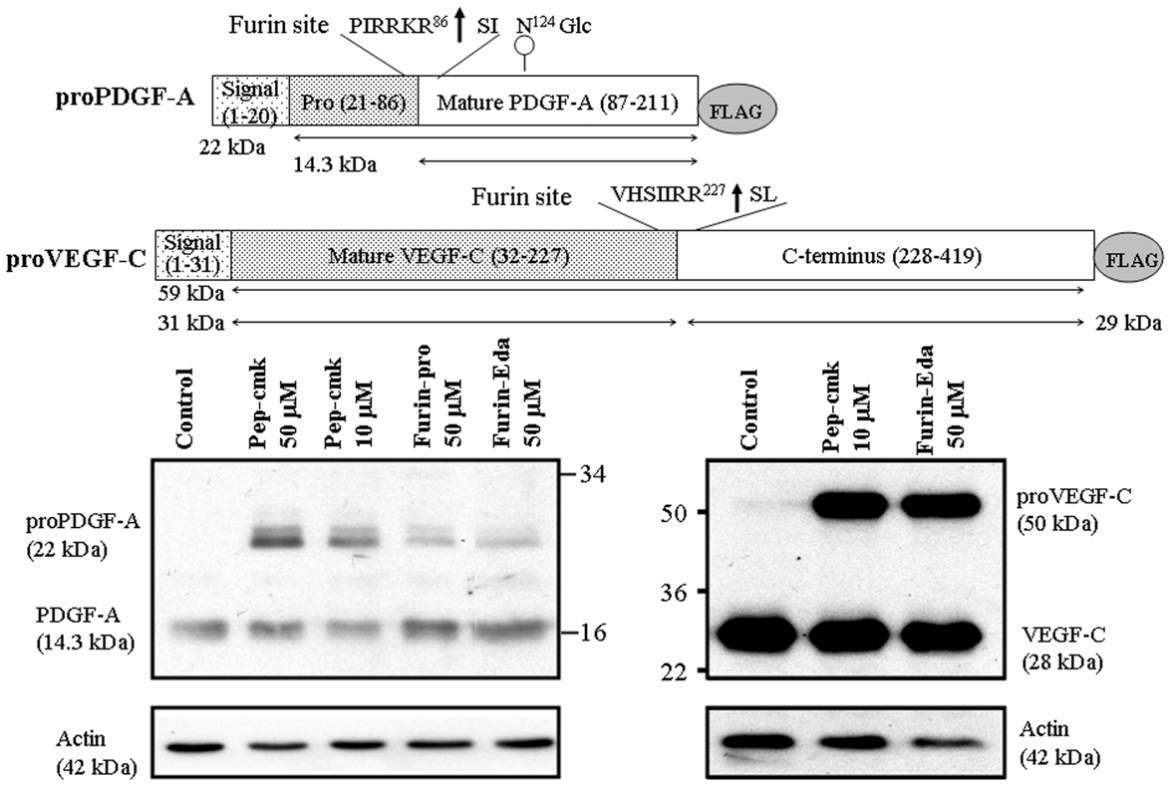
Schematic diagrams showing processing of hproPDGF-A and hproVEGF-C labeled at the C-terminus with a FLAG leading to their mature forms. The two upper panel figures highlight the furin processing sites (shown by vertical arrow) of the two precursor proteins. The lower panels show the effects of various furin inhibitors including the furin-Eda peptide (II) on the processing of proPDGF-A (left) and proVEGF-C (right) in CHO cell lines using western blot analysis. Pep-cmk  =  Dec-RVRR-cmk (chloromethyl ketone), Furin-pro  =  Synthetically made 83-mer full length hfurin prodomain (hfurin^25–107^) [49]. Actin levels were measured by western blots and used as controls for quantitation purpose.

Fig. 6 (left lower panel) showed the western blot results using FLAG antibody on the effect of furin-Eda-peptide (50 µM) on furin processing of FLAG-labeled proPDGF-A in CHO cells. The result was compared in parallel with the commercial furin inhibitor Dec (decanoyl)-RVRR-cmk (chloromethyl ketone) [48] and the 83-mer synthetic full length prodomain protein of hfurin [49]. Like these two inhibitors, our Eda-peptide was also able to block efficiently the cleavage of 22 kDa pro-PDGF-A to 14 kDa mature PDGF-A (Fig. 6, upper panel). This was reflected by the strong appearance of 22 kDa proPDGF-A band which was completely absent in the control experiment done in parallel without the presence of any inhibitor. In addition Fig. 6 (lower panel, right) showed by western blot using VEGF-C antibody, the effect of furin-Eda-peptide (50 µM) on furin-mediated processing of proVEGF-C in CHO cells. The effect was compared in parallel with that done with the known furin inhibitor dec-RVRR-cmk. The figure illustrated that like dec-RVRR-cmk, 50 µM of furin-Eda peptide was able to block significantly the cleavage of 50 kDa proVEGF-C to 28 kDa mature VEGF-C, confirming its furin inhibitory activity under *ex vivo* condition.

### Effect of Furin-Eda-Peptide on proPDGF-B Processing

**Figure 7:**
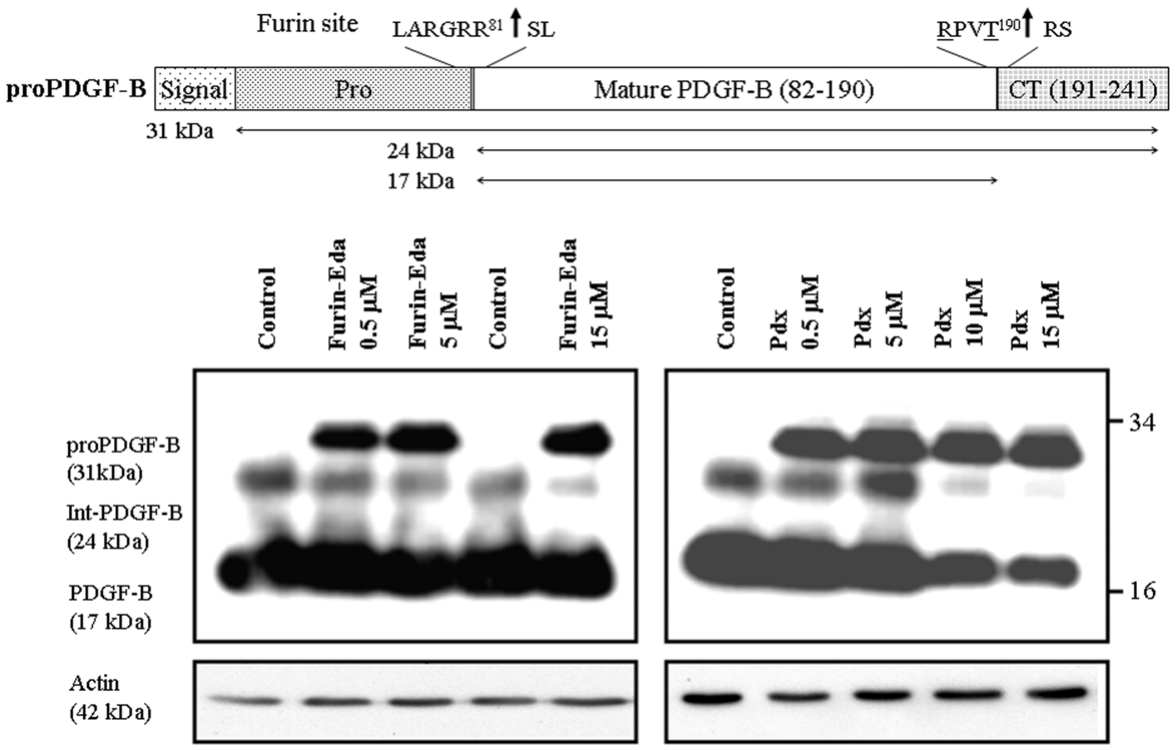
Schematic diagram showing proteolytic processing of hproPDGF-B leading to its mature form. Upper panel highlights the two processing sites including one by furin that lead to the production of its functionally active mature form; Lower panel: The lower panel shows the effects of Eda peptide (II) (left) and α1-Pdx (right), a known furin inhibitor on the processing of proPDGF-B in CHO cells at various concentration levels (5–15 µM) as indicated. Actin levels were measured by western blots and used as controls for quantitation purpose.

Furin-Eda-peptide (II) was also able to block furin-mediated processing of 31 kDa pro-PDGF-B to 17 kDa mature form in CHO cells in a concentration-dependent manner (Fig. 7). The results were highly comparable with those conducted in parallel with α1-Pdx protein, another known potent inhibitor of furin [50]. It is interesting to note that neither inhibitors displayed any effect on the formation of 22 kDa intermediate form of PDGF-B - whose exact identity as well as biological and functional role has not yet been fully explored.

### Interaction between Eda-Peptide and Recombinant Furin

**Figure 8:**
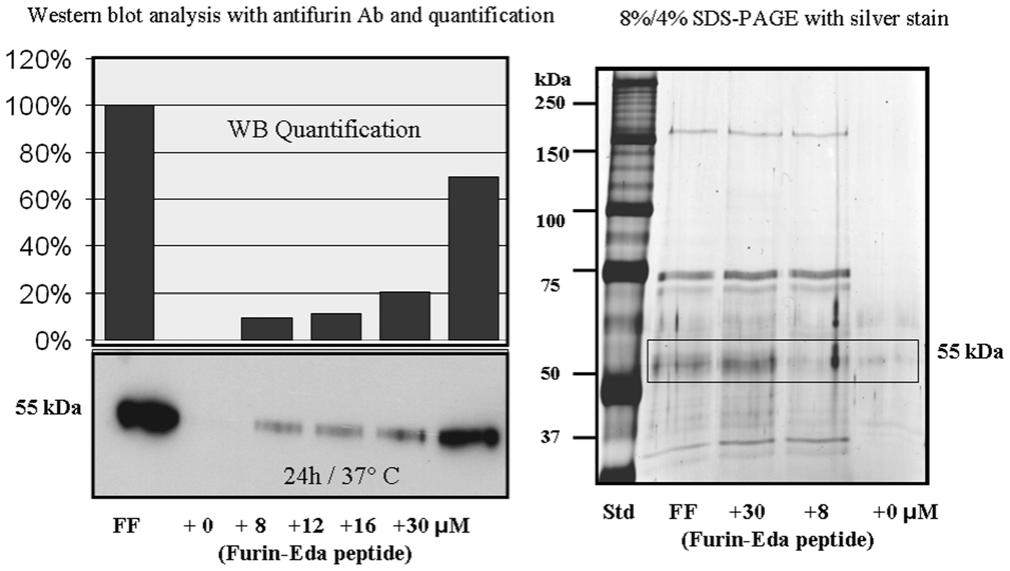
Western blots and SDS-gel electrophoresis with silver staining of fresh recombinant furin sample and samples after 24 h incubation in the absence and presence of various concentrations of furin-Eda-peptide (II). Left panel: Immunoblot analysis of various furin samples using furin-specific antibody. Right panel: Silver stains of same samples in SDS-gel electrophoresis for visualization of all protein bands. The 55 kDa band shown within a box represents the soluble form of recombinant furin protein. FF  =  Fresh furin sample; std  =  Standard.sample, Ab  =  Antifurin antibody.

In order to understand the plausible mechanism of furin inhibition by furin-Eda-peptide (II), recombinant furin protein was incubated with the Eda-peptide at various concentrations at 37°C for 24 h and the mixture was analyzed for protein bands by silver staining and western blots using anti-furin antibody. The results were compared with those of recombinant furin samples fresh as well as incubated in the absence of Eda-peptide. Western blot data (Fig. 8, left lower panel) indicated that Eda-peptide protected furin from self-degradation as the 55 kDa soluble furin band gradually became more intense as the concentration of Eda-peptide was increased. The results were also depicted by densitometric analysis of the observed bands (Fig. 8, left upper panel). A similar conclusion was also reached by protein staining data of the bands with silver ions (Fig. 8, right panel), which exhibited more intense 55 kDa furin band in the presence of higher concentration of Eda-peptide.

### 3D Model Structure of Furin-Eda Peptide

**Figure 9:**
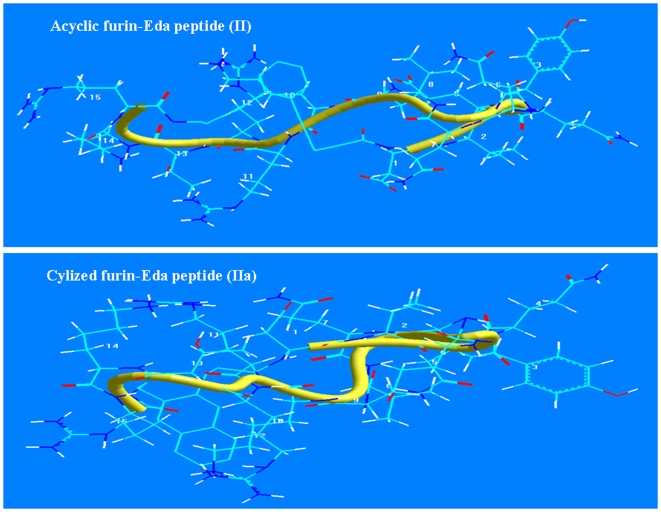
3D model structures of acyclic (II) and cyclic (IIa) furin-Eda-peptide based on hyperchem. The structures were generated by energy minimization using hyperchem program (version 7.5). The peptide backbones were shown in thick yellow lines.

Energy minimized 3D model structures of furin-Eda-peptide before and after Bergman cyclo-aromatization reaction were computed using hyperchem program and the structures were depicted in Fig. 9. It showed that the cyclic form of furin-Eda-peptide (IIa) exhibited a significant change in the backbone conformational geometry particularly at the C-terminal end of the molecule compared to the corresponding acyclic form (II).

### Docking of Furin-Eda-Peptide with the P-Domain of Furin Enzyme

**Figure 10:**
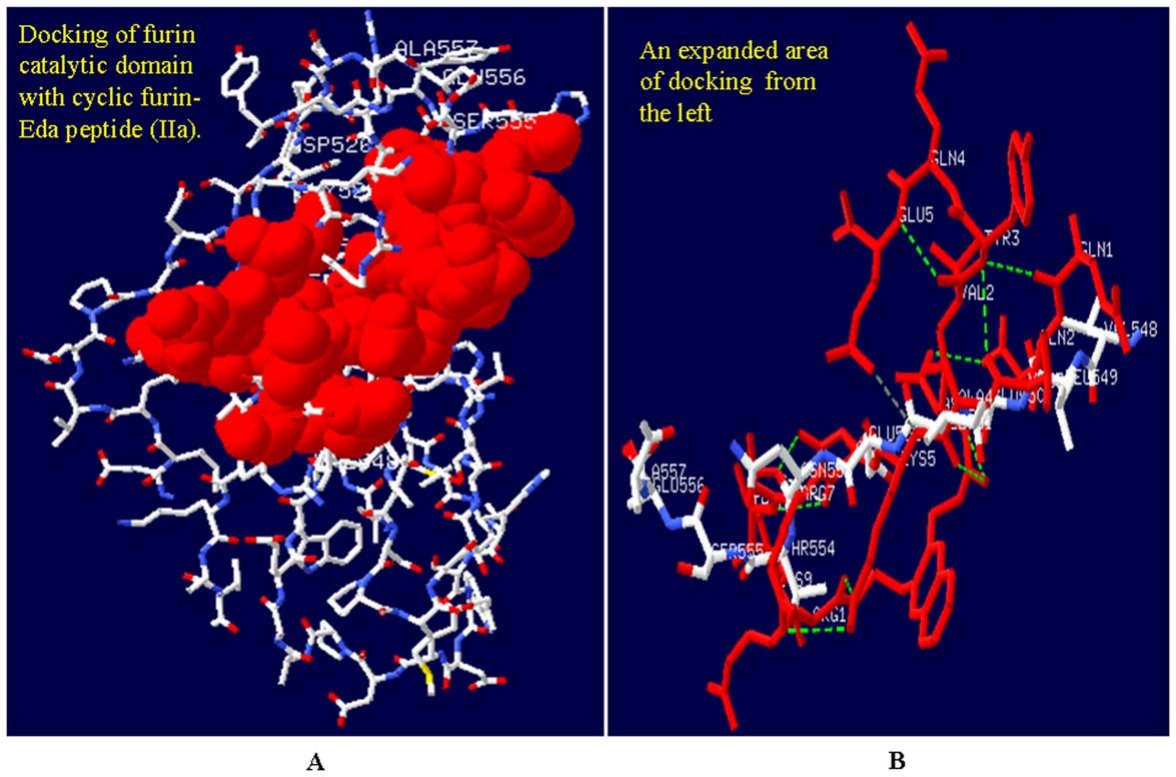
Interaction of furin-Eda peptide (II/IIa) with furin enzyme. Figure 10A. Docking of cyclized furin-Eda peptide (IIa) with furin P-domain (residue 487–560). Cyclized furin-Eda-peptide (IIa) is shown in space filled structure in red while the furin P-domain (residue ^487^Ala-Gln-Ala-Arg---------Ala-Asn-Asn-Tyr^560^) is depicted in stick mode and CPK (Corey, Pauling, Koltun) colors. This is the best fit docking structure obtained between the cyclic furin-Eda peptide (IIa) and any part of furin protein. Figure 10B. An expanded area of docking of furin P-domain with cyclized furin-Eda peptide (IIa) (shown in red). An enlarged segment of P-domain of furin characterized by the sequence ^548^VLEIENTSEA^557^ showing its strong interaction with cyclized furin-Eda-peptide (IIa) (shown in red). Several observed strong H-bondings between the two segments were shown by dotted lines.

Enediynes are known to undergo Bergman cyco-aromatization reaction even under mild condition (32). We therefore postulate that the observed inhibition of furin activity by furin-Eda-peptide may be mediated by the cyclic (IIa) rather than the acyclic (II) form. Our postulate is based on the speculation that the potency of binding will be increased upon cyclo-aromatization reaction due to increased π-stacking interaction. Further work using proton NMR spectroscopy would be necessary to provide more convincing evidence for the above hypothesis [51]. However owing to above proposition, docking studies were performed between various domains of furin particularly its P-domain and the cyclized furin-Eda-peptide (IIa) using autodock program. The results were shown in Figs. 10A and 10B (expanded) which revealed an efficient docking and potent interaction with the C-terminal segment of furin within the residues **^487^AQAR----------ANNY^560^**. A more detail investigation revealed strong H-bond interactions among several residues within the domain **(Val^548^---------Glu-Ala^557^)** and the cyclized furin-Eda peptide (Fig. 10B, enlarged portion). In fact our docking results identified a small segment **^548^VLEIENTSEA^557^** that interacts best with the cyclized Eda peptide (IIa). No significant interactions were noticed with the acyclic furin-Eda-peptide (II) suggesting the possibility that (IIa) might be the species responsible for interaction with furin as indicated above and this may ultimately lead to inhibition of furin activity which is composed of crucial catalytic residues D^153^, H^194^, and S^368^ as well as the oxyanion N^295^.

### Circular Dichroism (CD) Spectra of Eda-Peptide

**Figure 11:**
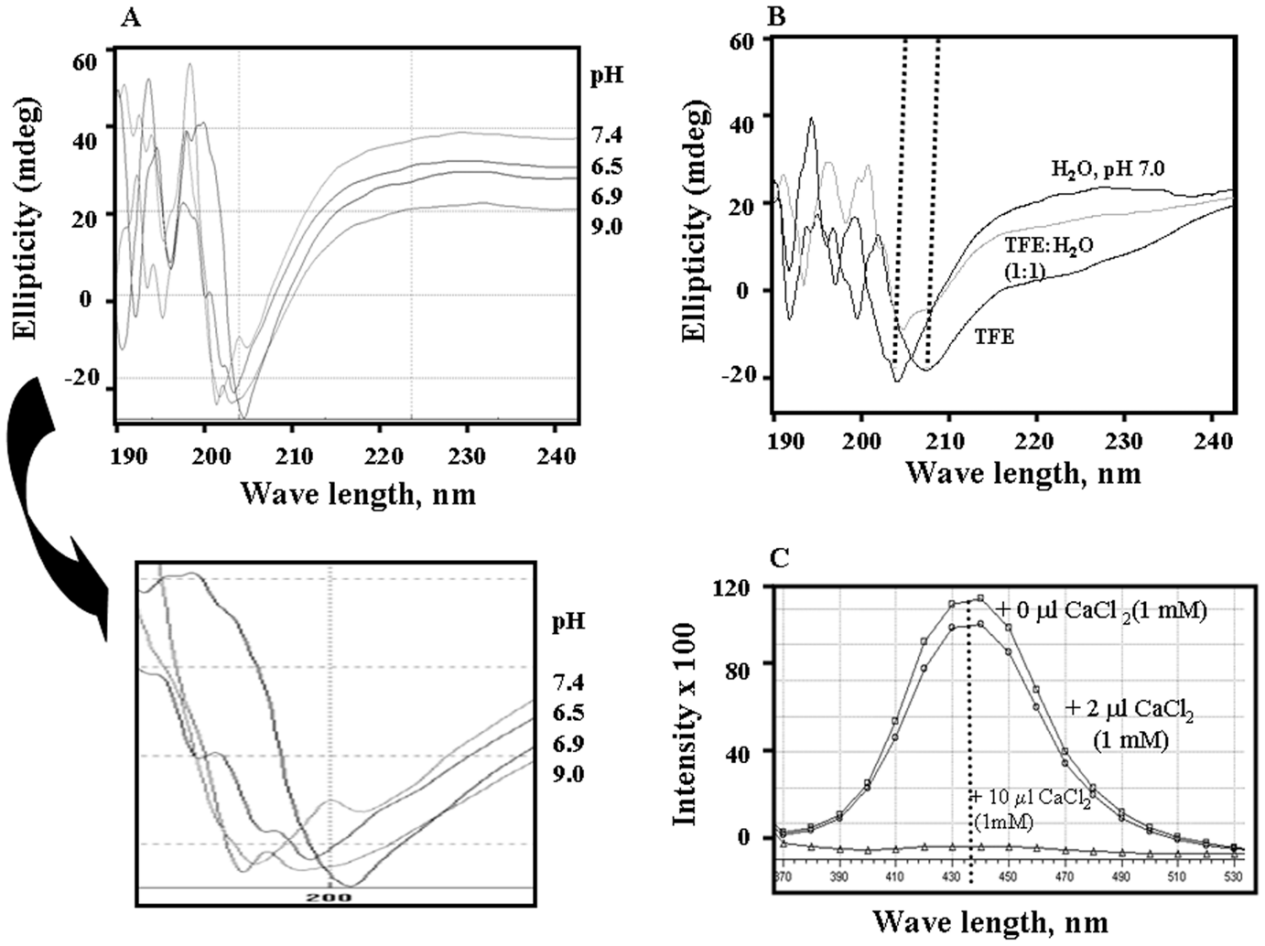
Circular dichroism and fluorescence spectra of furin-Eda peptide (II). Figure 11A. Overlay of Circular Dichroism (CD) spectra of furin-Eda-peptide (II) (0.5 mg/ml) in water at various pHs as shown. An expanded portion of the spectra showing the effects of pH on the position of the peak minima was shown directly beneath the full spectra. Figure 11B. Overlay of CD spectra of furin-Eda-peptide (II) (0.5 mg/ml) in various solvent systems as indicated. The gradual conversion of the spectra to more helix rich structure characterized by the presence of a maximum at ∼195 nm followed by two broad minima at ∼205 and ∼222 nm are noticeable. Figure 11C. Emission spectra (λ_ex_ = 320 nm) of Eda-peptide (II) in tetrafluoro ethanol (TFE) at various CaCl_2_ concentrations. The spectra were recorded in TFE solvent (100 µl, peptide concentration  = 0.1 mM)) in the absence. Addition of CaCl_2_ to the medium quenches the fluorescence intensity in a dose dependent manner. 1 mM CaCl_2_ can fully suppress the fluorescence as shown by the spectrum.

Circular dichroism (CD) spectrum [52] was conducted to evaluate the secondary structure of furin-Eda peptide which might play a role in its furin-inhibitory activity. The CD spectra of furin-Eda peptide in water at various pH conditions were depicted in Fig. 11A. The spectra showed that the peptide exists mostly in beta-sheet and random structures with very little helix content (<10%) under all pH conditions (5.5–8.0) tested. There were slight changes in CD profile particularly at 200–205 nm area following changes in pH condition. This may possibly due to partial transformation of sheet structure to random or turn structure. Addition of fluorinated alcohol such as TFE (a helix promoting solvent) led to the formation of significant helical structure at the expense of its sheet structure (Fig. 11B). This observation suggested that TFE can promote helix structure in furin-Eda-peptide which might alter its furin inhibitory property.

### Fluorescence Photophysical Property of Eda-Peptide

Since Eda-peptide contains aromatic and conjugated double and triple bonds in its structure we decided to examine its fluorescence spectroscopic property. In fact it was found to exhibit excitation and emission peaks at 320 and 440 nm respectively (*data not shown*). The emission spectra of furin-Eda-peptide in TFE in the absence and presence of various concentrations of CaCl_2_ which is required for furin activity were shown in Fig. 11C. The graph showed that upon addition of CaCl_2_ the peak at 440 nm observed in the emission spectrum gradually diminished depending on the amount of added CaCl_2_. In fact the peak was completely suppressed upon addition of equimolar quantity of CaCl_2_, suggesting a strong interaction between furin-Eda-peptide and CaCl_2_. A similar conclusion was also reached from CD spectra studies (*not shown*).

## Discussion

This study revealed for the first time that fully nonpeptide aromatic and heterocyclic enediyne compounds can inhibit furin activity *in vitro* with IC_50_ in low to medium micromolar ranges depending on the nature of the structure and type of substituent present. Enzyme inhibitory activity was also noticed towards other PC enzymes namely PC5 and PC7 (Fig. 1). Among the compounds tested, a 12-membered 1, 4-diaza enediyne heterocyclic ring compound with bis-benzyl substituent (AB-2) has been found to exhibit highest inhibitory activities against both furin and PC7 with IC_50_ of 8.5 and 10.5 µM respectively. However it was found to be a less potent inhibitor towards PC5, with IC_50_ of 34 µM. Thus clearly there was a lack of selectivity for inhibition when compared among these PCs. The next best furin inhibition was achieved with the compound AB-3 followed by AB-1. Interestingly while both AB-3 and AB-4 contain a common 13-membered 1–3 diaza ring structure, AB-1 is an acyclic molecule. The tetra aza compounds AB-6 and its Nickel-complex were found to be much poor inhibitors of furin.

In addition to above, our study for the first time showed that an enedynyl peptide can inhibit the protease activity of a PC enzyme such as furin. However while this manuscript is under review, the inhibition of chymotrypsin by an enediynyl peptide was reported in the literature [53]. Thus here we revealed that incorporating an enediynyl “Eda” function - a highly reactive and beta turn inducing amino acid [44] at the junction of P1-P1′ residues of a furin substrate derived from its prodomain primary catalytic site, led to a highly potent furin inhibitor. Other properly designed unnatural amino acids may also be incorporated instead of “Eda” to design other furin inhibitors. In the present design as our first choice, the prodomain sequence comprising (98–110) amino acid residues of human preprofurin was selected as the substrate. The significance of prodomain as well as its removal and or degradation during furin activation have been well documented in the past [2], [3], [5] and more recently in [54]. The presented data based on progress curve, Dixon plot and sigmoidal graph (Fig. 5) all indicated that furin-Eda-peptide (II) is a strong inhibitor of Proprotein Convertase furin. Furthermore the data confirmed the competitive nature of inhibition which was also supported by CD and fluorescence studies. The data also suggested that the furin inhibition by Eda-peptide is independent of the nature of the substrate used – be it a small peptide or a physiological protein. In fact (II) inhibited furin cleavages of hSARS coV peptide [55] as well as physiological protein substrates such as growth factor precursers. Although in the present study “Eda” was introduced between P1/P1′ amino acids, it will be more logical to replace or substitute P1′ residue by “Eda” which is expected to lead to even more potent and/or selective inhibitor of furin. Besides, other peptides substrates of furin derived from the processing site of its physiological proproteins can be employed to validate this new hypothesis of ours for protease inhibitor design. These aspects are currently being investigated in the laboratory.

Our studies involving cell lines expressing furin and its physiological substrates proPDGF-A, proVEGF-C and proPDGF-B indicated that Eda-peptide when added exogenously into the culture media during cell growth can efficiently block furin-mediated processing of above substrates leading to their bio-active mature forms in a concentration dependent manner. In most cases the effects with our furin-Eda peptide were comparable to those observed with the known furin inhibitors α1-Pdx [50] and Dec-RVRR-cmk [48]. In other cases the effects were slightly less pronounced but still significant. Thus for example western blot results showed that 50 µM of Eda-peptide blocked furin-mediated processing of proVEGF-C to mature VEGF-C to a similar extent compared to that obtained with 10 µM Dec-RVRR-cmk (Fig. 6). Similar observation was also noted for proPDGF-A processing. However, the Eda peptide was found to be more effective in blocking the cleavage of proPDGF-B. In fact, both Eda-peptide and α1-Pdx suppressed the cleavage of proPDGF-B to its mature form almost with identical efficiency (Fig. 7). Thus both *in vitro* and *ex vivo* studies involving small peptide as well as natural protein substrates confirmed the potent anti-furin activity of Eda-peptide (II). The observed K_i_ and IC_50_ values for Eda-peptide as measured against Boc-RVRR-MCA were found to be higher than that observed with bioengineered protein α1-Pdx [50] but were still in low nM range. Our studies confirmed that Eda-peptide can block furin cleavage of fluorogenic peptides as well as physiologically relevant proteins proPDGF-A, B and proVEGF-C. Proteolytic activation of these growth factor precursor proteins has been linked to tumor growth and progression as shown by the high level of expression of their mature forms in cancer cells compared to normal ones [7], [56]–[58]. Thus above research findings suggested that our Eda-peptide may play an important role in tumor suppression and intervention via the blockade of maturation by furin of precursor growth factor proteins. More potent and selective furin inhibitors based on “Eda” may be developed by choosing more efficient furin recognition sequences based on its known substrates.

Secondary structure analysis by circular dichroism spectra indicated that the Eda-peptide exhibited predominantly sheet and random structures with little content of helical structure in aqueous medium under physiological pH condition (pH 7.4). Change in pH has little effect on the secondary structure, although the spectrum profile at the minima showed significant differences. Moreover, addition of helix promoting solvent such as TFE led to a noteworthy increase in helix structure thereby suggesting that Eda-peptide is capable of adopting helix conformation depending on the nature and pH of the solvent. This structural behaviour is partly consistent with that found in the similar region of furin protein as revealed by its x-ray crystal structure [59], [60].

Molecular docking studies indicated that upon Bergman cyclization furin-Eda-peptide provided a more potent interaction and better contact with the P-domain of furin protein characterized by the segment ^548^Val-Leu-Glu-Ile-Glu-Asn-Thr-Ser-Glu-Ala^557^ which is located not too distant from the catalytic domain [61]. The complete catalytic domain of furin (108–438) is defined as the sequence segment beginning at the mature N-terminus and ending at the residue equivalent to the C-terminus of thermitase [61]. The above binding may lead to a significant change in conformation and folding pattern of furin catalytic structure leading to its loss of protease activity. No other docking could be noticeable between the Eda-peptide (cyclic or acyclic form) and any domain of furin. Thus, it is more likely that Eda-peptide upon Bergman cyclization may be a more effective inhibitor of furin although such conclusion would require further studies with the two purified forms of furin-Eda peptide and NMR Spectra. Moreover, in order to further expand the present study and examine the efficacy of Eda-peptide approach as a general method for inhibitor design for any protease including furin, other peptide sequences need to be examined. Moreover further studies in future using additional cell lines and animal models will shed more light on the mechanism of furin inhibition by Eda-peptide.

## Materials and Methods

### Materials

All Fmoc-protected amino acid derivatives (L-configuration), coupling reagents, resins for peptide synthesis as well as the fluorogenic substrate Boc-RVRR-MCA and furin inhibitor Dec-RVRR-cmk were purchased from Bachem Inc (King of Prussia, Pa, USA), Calbiochem Novabiochem Inc (San Diego, Ca, USA), Neosystems Inc, (San Diego, Ca, USA) or PE Applied Biosystems (Foster City, Ca, USA). The primary and secondary antibodies for PDGF-B, Actin and FLAG tag were bought from Santa Cruz Biotechnology Inc (Santa Cruz, Ca, USA) whereas all reagents for immuno-blotting and SDS-PAGE were purchased from Biorad Labs (Hercules, Ca, USA). Surface Enhanced Laser Desorption Ionization and Matrix Assisted Laser Desorption time of flight mass spectra were recorded using Ciphergen Protein Chips (San Diego, Ca, USA) and Voyageur DE Pro (PE-Applied Biosystems, Framingham, Ma, USA) respectively. The energy absorbing matrices α-cyano 4-hydroxy cinnamic acid and 1, 2-dihydroxy benzoic acid and silica gel 60 for column chromatography were purchased from Sigma Chemical Company (Mississauga, On, Canada) and Sigma-Aldrich Chemical Company (St Louis, Mo, USA) respectively. The solvents used were HPLC grade and were purchased from Fisher Chemical Company, Mississauga, On, Canada. RP-HPLC was performed on C_18_ column (analytical and semi-preparative) using Varian instrument and Star software program. The gradient used was as described in [27].

### Synthesis of Aryl Enediyne Compounds AB1-6

Chemical syntheses and full characterizations of various aryl enediyne compounds **AB1**, **2**, **3**, **4**, **5**, **6** and **6**-**Ni** listed in Fig. 1 were accomplished previously using multiple chemical steps as described in [32].

### Synthesis of Fmoc-Enediynyl Amino Acid (Eda) (I)

Fmoc enediynyl amino acid (Eda) was synthesized using several steps as described in (42). In brief, the synthesis was accomplished first by Pd (0)-mediated Sonogashira coupling of 1, 2-dibromo benzene with 4-pentynoic acid benzhydryl ester in presence of cuprous bromide in dry triethylamine under refluxing condition for 4 h in an inert atmosphere. After aqueous work up, the crude oily residue was purified by column chromatography using silica gel 60 (eluted with petroleum ether: Ethyl acetate  = 30∶1) to afford the eneyne ester [5-(2-Bromo-phenyl)-pent-4-ynoic acid benzhydryl ester]. A second round of Sonogashira coupling of eneyne ester with 3-butyne-1-ol and subsequent purification yielded the enediyne hydroxyl ester [5-[2-(4-Hydroxy-but-1-ynyl)-phenyl]-pent-4-ynoicacid benzhydryl ester]. Thus enediyne hydroxyl ester obtained was converted to methyl toluene sulphonate or mesylate and was subsequently reacted with sodium azide in dry DMF to furnish the corresponding enediyne azido ester [5-[2-(4-Azido-but-1-ynyl)-phenyl]-pent-4-ynoic acid benzhydryl ester]. The azido functionality was then reduced to amine on treatment with triphenyl phosphine and H_2_O in THF. The amine [5-[2-(4-Amino-but-1-ynyl)-phenyl]-pent-4-ynoic acid benzhydryl ester] was protected with Fmoc by reaction with Fmoc-chloride under basic condition in methanol to afford Fmoc-NH-Eda-benzhydryl ester which was then subjected to deprotection by TFA/anisole in dry dichloromethane to furnish the desired Fmoc-Eda (I), [5-[2-(4-Fmoc-amido-but-1-ynyl)-phenyl]-pent-4-ynoic acid] whose calculated molecular weight (449) was found to be in good agreement with the observed molecular weight (450).

### Synthesis of Furin-Eda Peptide (II)

Peptide synthesis was accomplished by Fmoc-based solid phase chemistry (0.25 mmol scale) using Fmoc-PAL-PEG-PS (polyamino linker polyethylene glycol) resin, substitution  = 0.55 meq/gm resin). [PAL  =  [{5-(4-amino methyl 3.5-dimethoxy phenoxy) valeric acid, PEG  =  polyethylene glycol and PS  =  polystyrene resin)] and HATU (O-hexafluoro-phospho-[7-azabenzotriazol-1-yl]-N, N, N', N'-tetramethyluronium)/DIEA {di-isopropylethylamine} coupling agent (30). Following side chain protecting groups were used for various Fmoc protected amino acids: t-butyloxy carbonyl (tBoc) for Lys; tertiary butyl (tBut) for Thr, Tyr, Asp, Glu, (2, 2, 4, 6, 7-pentamethyl dihydrobenzofuran-5-sulfonyl) (Pbf) for Arg and finally trityl (Trt) for Gln. Following completion of synthesis, the crude peptide was cleaved from the resin and fully deprotected with Reagent B [26], [27].

### Purification and Characterization of Peptide (II)

The crude peptide (II) as obtained above was purified by RP-HPLC using semi-preparative and analytical C_18_ columns in combination. Peaks were collected and examined by mass spectrometry. The peak eluting at retention time (Rt)  = 26.5 min was characterized as furin-Eda peptide (II) by both SELDI and MALDI-tof mass spectrometry using CHCA matrix [MW calculated  = 2110, observed 2111 (M^+^+H), 2128 (M^+^+H+O.) and 2133 (M^+^+H+Na)].

### Source of Enzymes

While pure soluble recombinant h (human) furin was obtained commercially from New England Biolabs (Boston, Ma, USA), recombinant m (mouse) PC5 and soluble hPC7 were obtained in partially purified form as described earlier [18].

### Determination of Kinetic Parameters, K_i_ and IC_50_ for Inhibition of Furin by Eda Peptide (II)

Inhibition constant K_i_ for inactivation of furin by Eda peptide (II) was determined by Dixon plot using three different concentrations of substrate **Boc-RVRR-MCA** and various concentrations of the inhibitor ranging from 0–300 nM. For IC_50_ determination, a sigmoidal graph was generated by plotting the reaction rate as measured by raw fluorescence unit (RFU) released per hour against the logarithm of concentration of inhibitor. In case of latter, the substrate used was an intramolecularly quenched fluorogenic peptide, **Abz-AEQDRNTR^761^↓EVFAQ-Tyx-A** (Abz  = 2-Amino benzoic acid, Tyx  = 3-Nitro tyrosine) derived from residues {754–766} of hSARS corona virus spike glycoprotein as previously reported [8].

### Effect of Furin-Eda Peptide on the Processing of proPDGF-A in CHO Cells

CHO-cells expressing FLAG-tagged PDGF-A were grown in DMEM medium as described (43) until the cells achieved ∼80% confluency (24 h), At this stage the medium was removed, cells were washed and grown in fresh buffer supplemented with furin-Eda peptide (50 µM final concentration). The media and cells were harvested after another 24 h cell growth. The collected media were concentrated ∼20-fold using centricon filtration system (Amersham Biosciences Inc, cut off MW  = 3 kDa) and subjected to western blot analysis using FLAG antibody for the presence of full length proPDGF-A and its C-terminal mature form obtained following cleavage by endogenous furin present in CHO cells. In parallel, identical experiments were performed by growing cells in the absence or presence of known furin inhibitors namely dec-RVRR-cmk (10 and 50 µM concentrations) and synthetic 83-mer full length furin-prodomain protein (50 µM final concentration), previously described by us [49]. For quantitative purpose, each sample was also analyzed for the presence of actin by immunoblot using a specific antibody.

### Effect of Furin-Eda Peptide on the Processing of proVEGF-C in CHO Cells

CHO-cells expressing FLAG-labeled proVEGF-C [57] were grown in DMEM medium as described above in the absence and presence of furin-Eda peptide and the furin inhibitor Dec-RVRR-cmk (each 50 µM concentration). The presence of VEGF-C precursor and its C-terminal processed form generated by furin cleavage were assessed by western blot analysis using FLAG antibody while the level of actin (control house keeping protein) was analyzed by immunoblot for quantitation purpose.

### Effect of Furin-Eda Peptide on the Processing of proPDGF-B in CHO Cells

CHO-cells expressing endogenous proPDGF-B [58] were grown as described above in the absence and presence of various concentrations ranging from 0.5 to 15 µM of either furin-Eda peptide or α1-Pdx, a known furin inhibitor [50]. Western blots were performed for the presence of proPDGF-B and its processed form using PDGF-B specific antibody. In parallel samples were also analyzed for actin levels.

### Effect of Furin-Eda Peptide on Recombinant Furin Protein

Recombinant C-terminal truncated soluble furin [2 µl, activity  = 4U (unit), MW 55 kDa, 1 U of activity releases 1 pmol of free AMC from 100 µM fluorogenic Boc-RVRR-MCA substrate in one minute at 37°C] was incubated for 24 h at 37°C in 20 mM Tris +25 mM Mes +2.5 mM CaCl_2_, pH 7.4 in the absence or presence of furin-Eda-peptide (II) at various concentrations ranging from 0–30 µM. Samples were analyzed by western blot (using furin antibody from SantaCruz) as well as silver staining for the presence of all protein bands.

### Circular Dichroism (CD) Spectra of Furin-Eda (II) Peptide

All CD spectra (run in triplicates and then averaged) were recorded at 25°C in Jasco-810 spectropolarimeter (Easton, Md, USA) in 0.1 cm thick rectangular quartz cell in a total volume of 300 µl (100 µM final concentrations) at 0.1 nm intervals from 185–240 nm wave length. The final corrected CD spectra were obtained by subtracting the spectrum of the control solvent run in parallel from that of the sample spectrum and analyzed using CD. Estima and Contin software programs for α-helix, β-pleated sheet, β-turn, and random structures were used (Softspec Company, NJ, USA) [52].

### Fluorescence Spectrum of Furin-Eda Peptide (II)

The fluorescence emission spectrum of furin-Eda peptide (0.1 mM) was recorded at a fixed excitation wave length 320 nm in a well plate in tetrafluoro ethanol (TFE) solvent (100 µl) at ambient temperature in absence or presence of added (2 or 10 µl) CaCl_2_ solution.(1 mM stock solution).

### Molecular Model of Furin-Eda-Peptide Before and After Bergman Cyclo-Aromatization

Theoretical 3D molecular model structures were generated for Eda-peptide both acyclic (II) and cyclic (IIa) forms by using Hyperchem software (version 7.5, Hypercube Inc) based on the energy minimization using Polak-Ribiere algorithm program.

### Docking of Furin-Eda-Peptide with Furin

Docking of Eda-peptide, both acyclic and cyclic forms with the catalytic domain of hfurin (sequence accession number  =  NP_002560) based on its known crystal structure (pdb file 1p8j) [59], [60] was accomplished by using autodock and modeler programs (for comparative protein structure modeling by satisfaction of spatial restraints) (http://salilab.org/modeller/).
